# Editorial: Embodied neuromorphic AI for robotic perception

**DOI:** 10.3389/frobt.2026.1956418

**Published:** 2026-08-19

**Authors:** Jorge Dias, Fakhreddine Zayer, Mario Molinara

**Affiliations:** 1 Center for Autonomous Robotic Systems (CARS), Khalifa University, Abu Dhabi, United Arab Emirates; 2 Institut National des Technologies et des Sciences du Kef, El Kef, Tunisia; 3 Khalifa University, Abu Dhabi, United Arab Emirates; 4 Department of Electrical and Information Engineering, University of Cassino, Cassino, Italy

**Keywords:** artificial intelligence, embodied AI, neuromorphic computing, robotic perception, robotics

## Background

This Editorial is a summary of articles on Neuromorphic Computing for Robotics and AI. This Research Topic brings together extended contributions associated with the Embodied Neuromorphic AI for Robotic Perception workshop held at the 2023 IEEE International Conference on Robotics and Automation (ICRA), together with additional relevant submissions. The Research Topic examines how neuromorphic computing, event-based sensing, compact learning systems, and resource-aware artificial intelligence can support a new generation of autonomous robotic agents.

Embodied neuromorphic AI seeks to couple perception, learning, prediction, planning, and motor control within agents that operate directly in complex and uncertain environments. Unlike conventional approaches that often improve performance by adding general-purpose computation, redundancy, and control structures, neuromorphic systems aim to achieve robustness with substantially lower power, memory, latency, and hardware overhead. This is especially important for mobile robots, autonomous ground vehicles, and unmanned aerial vehicles, whose operation is constrained by battery capacity, size, and real-time response requirements.

Intelligent behaviour also depends on processing information across multiple timescales: rapid sensory interpretation, hierarchical extraction of relevant features, and retention of temporally structured information for learning and adaptation. Conventional platforms can implement these functions, but generally at a cost far above that of biological neural systems. Neuromorphic engineering addresses this gap through mixed-signal analogue and digital hardware, event-driven sensors, and neural computational primitives inspired by biological intelligence. Important challenges remain, including the integration of custom neuromorphic chips with sensors, conventional processors, and actuators; the programming of neural systems on specialised hardware; and the development of principled methods for combining neural representations and computational operations.

This article is about uncertainty-aware sand and dust image restoration (Wei et al.). The first contribution introduces the Hierarchical Interactive Uncertainty-aware Network (HIUNet) for restoring single images degraded by sand and dust. Dusty scenes present heterogeneous and uncertain degradation that can limit the reliability of standard deep learning methods. HIUNet addresses this problem by using Bayesian neural networks to obtain robust shallow features, a frequency-based selection mechanism to retain useful information while suppressing redundancy, and a feature-enhancement module to refine the reconstructed image. Experiments on the Sand11K dataset, which contains varied degradation levels, demonstrate the effectiveness of the proposed framework and highlight the value of uncertainty modelling for robotic perception in adverse visual conditions.

This article provides an overview of model-compression techniques for practical deployment (Liu et al.). The second contribution surveys the development of model compression techniques and their role in deploying large and domain-specific models under practical hardware constraints. Large models require substantial training effort, memory, and computational capacity, making efficient inference difficult on embedded and edge platforms. The review examines quantisation, pruning, low-rank decomposition, and knowledge distillation, outlining their principles, recent progress, and deployment implications. By organising past and current approaches while identifying future research directions, the survey provides a technical reference for reducing model size and computational demand without abandoning application-level performance.

This article is about energy-efficient embodied spiking neural networks (Putra et al.). The third contribution presents SNN4Agents, a framework for designing energy-efficient embodied spiking neural networks for autonomous agents. Spiking neural networks can exploit sparse event-based computation, but systematic optimisation procedures for deployment remain underdeveloped. SNN4Agents combines weight quantisation, timestep reduction, and attention-window reduction to improve energy efficiency, memory use, and latency while maintaining accuracy. In event-based car recognition on the NCARS dataset, the framework achieves 84.12% accuracy together with 68.75% memory savings, a 3.58× speed-up, and a 4.03× improvement in energy efficiency relative to the referenced state-of-the-art method. These results illustrate the potential of coordinated optimisation for practical neuromorphic autonomy.

This article is about mapless navigation with self-supervised cognitive maps (Polykretis et al.). The fourth contribution addresses mapless navigation in unknown environments using a shallow architecture trained through a local learning rule. Deep reinforcement learning methods can achieve strong navigation performance, but multi-layer networks, complex reward objectives, and backpropagation increase memory and computational demands. The proposed method combines novelty-based exploration with random walks, reducing dependence on manually defined rewards and supporting greater agent autonomy. It reaches performance comparable to a deep Q-network in goal-reaching accuracy and path length while avoiding error backpropagation. Its shallow structure and local learning mechanism make it suitable for edge neuromorphic processors and resource-constrained robots.

## Final outlook

Together, the contributions show that embodied neuromorphic AI is not defined by a single algorithm or device. Progress depends on the joint design of sensing, representation, learning, model optimisation, hardware, and control. Robust perception under uncertainty, compressed models, efficient spiking networks, and local self-supervised learning are complementary steps toward autonomous agents that can operate for longer periods, react in real time, and adapt with limited resources.

Future work should strengthen integration across sensors, processors, and actuators; establish clearer programming and benchmarking frameworks; and evaluate complete systems in realistic, changing environments. Such coordinated efforts can help neuromorphic robotics move from specialised demonstrations toward dependable agents that interact naturally and safely with society.

